# Clinical and Radiological Outcomes of Titanium Elastic Intramedullary Nailing in Pediatric Tibial Shaft Fractures

**DOI:** 10.7759/cureus.103885

**Published:** 2026-02-18

**Authors:** Faheem Ullah Khan, Fayaz Hussain, Sher Baz Khan, Fayyaz Ur Rahman Haider, Syed Ahson Abbas, Shabbar H Changazi, Akbar Shah

**Affiliations:** 1 Orthopaedics and Traumatology, Provincial Headquarter Teaching Hospital, Gilgit, PAK; 2 Internal Medicine, Dudley Group National Health Service (NHS) Foundation, Dudley, GBR; 3 General Surgery, Provincial Headquarter Teaching Hospital, Gilgit, PAK; 4 Neurological Surgery, Provincial Headquarter Teaching Hospital, Gilgit, PAK

**Keywords:** bone nails, fracture healing, fractures, intramedullary nailing, pediatric, tibia

## Abstract

Background

Tibial shaft fractures are common injuries in children. Stable fractures are often managed non-operatively. Unstable fractures typically require surgical intervention. Titanium elastic intramedullary nailing (TEN) is a widely used minimally invasive technique. It provides stable fixation and promotes fracture healing through controlled micromotion. This study aimed to determine the mean union time for pediatric tibial shaft fractures treated with TEN.

Methods

We conducted a descriptive case series over three years. It included 68 children aged one to 15 years. All participants had closed, transverse tibial shaft fractures (Arbeitsgemeinschaft für Osteosynthesefragen/Orthopaedic Trauma Association (AO/OTA) 42-A3). We excluded patients with open fractures, segmental patterns, or significant comorbidities. All patients underwent closed reduction and internal fixation with two pre-bent titanium elastic nails. The primary outcome was the mean duration to union. Union was assessed clinically by pain-free weight-bearing and radiologically by bridging callus on at least three cortices.

Results

The mean participant age was 8.03 years with a standard deviation of 2.16 years. There was a male predominance (n=40, 58.82%). The overall mean union time was 12.41 weeks with a standard deviation of 2.30 weeks. Stratification analysis showed a significantly shorter union time in older children aged eight to 15 years (n=31, 45.59%) compared to younger children aged one to seven years (n=37, 54.41%). The union times were 11.61 versus 13.08 weeks, respectively, with a p value of 0.008. We observed no significant differences in union time concerning gender, patient weight, or time from injury to surgery.

Conclusion

TEN is an effective and reliable method for managing unstable tibial shaft fractures in children. It results in predictable union with a relatively short healing time. This minimally invasive procedure should be considered a standard option in pediatric orthopedic care.

## Introduction

Pediatric tibial shaft fractures are common and significant injuries. They represent 10%-15% of all long bone’s fractures in children [1]. The most frequent mechanism of injury is moderate to high-energy trauma, such as sports injury, falls, and motor vehicle accidents. Although most of these fractures can be successfully treated with nonoperative measures, including casting, a subset is associated with intrinsic instability. These are fractures with high-displacement, comminution, or length-unstable patterns. These fracture patterns are unstable and may have poor outcomes of malunions, delayed unions or prolonged immobilization when managed nonoperatively [2].

The treatment of these unstable fractures has evolved. The trend now is against prolonged immobilization and shifted towards surgical stabilization for proper anatomical alignment and early mobilization. Titanium elastic intramedullary nailing (TEN) has become a gold standard method for diaphyseal fractures in children among several surgical methods [3]. The underlying concept of this procedure is to obtain stable and elastic fixation. This is accomplished by symmetric insertion of two pre bent nails in the medullary canal forming a three-point support buttress. This construct serves as an inner splint. It maintains adequate stability for the healing period while allowing controlled micromotion at the fracture site. This micromotion serves as an important biological stimulus for the generation of a strong periosteal callus [4].

The advantages of TEN are well documented. They extend beyond its biomechanical efficacy. It is a minimally invasive and physeal sparing procedure that conserves the fracture hematoma and avoids excessive dissection of soft tissue. This results in a lower infection rate and better cosmetic aesthetic than open reduction and internal fixation [5]. In addition, it also allows early postoperative ambulation and shortens the period of recovery. This is especially advantageous in the paediatric age group. Recent comparative studies continue to support its superiority to other modalities, such as external fixation. These studies demonstrated that TEN is associated with quicker healing time and improved functional results, as well as higher patient satisfaction. This is due to the avoidance of pin site complications and the burden of external frames [6].

While TEN has become the standard treatment for paediatric tibial shaft fractures (PTSF) worldwide, its results are context dependent. The case of Far-Falong and beyond in the remote high-altitude Gilgit-Baltistan region of Pakistan is particularly special, both for its peculiarities and significance. This population is specifically challenged by reduced availability of immediate specialized orthopedic care, nutritional factors influenced by seasonally available food sources, and early-life high levels of physical activity in response to terroir and agrarian lifestyle. Second, the geographic remoteness may affect compliance with follow-up and rehabilitative measures. Although union times of 10-13 weeks have been described in the worldwide literature [7,8], such references may not be directly applicable to this particular setting where factors such as altitude or socioeconomic and localized clinical practices could influence the healing process. Therefore, the primary objective of this study is to determine the mean duration of union for unstable tibial shaft fractures in children treated with titanium elastic intramedullary nails at local setup. This will contribute contemporary and relevant data to the existing body of evidence.

## Materials and methods

This descriptive case series was conducted at the Department of Orthopedics, Provincial Headquarter Teaching Hospital Gilgit, over a three-year period, from December 2022 to November 2025. We enrolled a total of 68 consecutive patients who presented with closed tibial shaft fractures and met the inclusion criteria. The sample size was calculated as 68. This calculation used a 95% confidence level and 1% desired precision, based on a mean union time of 11.17 weeks with a standard deviation of 2.81 weeks from a previous study [9]. We included patients aged 1 to 15 years of either gender with a closed, transverse tibial shaft fracture (Arbeitsgemeinschaft für Osteosynthesefragen/Orthopaedic Trauma Association (AO/OTA) 42-A3) presenting within seven days of injury. Exclusion criteria included a history of prior surgery on the affected fracture, segmental fractures, open or infected wounds, refusal of consent by the patient's guardian, and the presence of chronic liver or renal disease (effectively excluding pathological fractures).

Ethical approval was obtained from the institutional review board and informed consent taken from parents or guardians. All procedures were performed under general anaesthesia. The patient was positioned supine on a radiolucent table. The fracture was reduced under the c-arm. The ideal nail size was preoperatively defined as 40% of the smallest diameter of canal. The titanium elastic nails were pre-bent in 'C' shape. We adopted a medial proximal tibial approach. A 2-cm incision was then made distal to the proximal tibial physis. A bone awl was used to make the entry. The nail was then gently inserted into the medullary canal and fracture site. A second nail was inserted laterally to achieve symmetrical fixation. All procedures were performed by a consultant orthopedic surgeon with at least three years of post fellowship experience in the field.

Postoperative clinical and radiological evaluations were conducted weekly for three weeks, biweekly for six weeks, and fortnightly for 15 weeks. Fracture union was defined as a combination of clinical and radiographic criteria. Clinical union was defined as the ability to bear full weight without pain (visual analog scale (VAS) score ≤1) and perform a painless single-leg stance using the fractured limb. Radiographic union was defined as bridging callus across at least three of four cortices on standardized anteroposterior and lateral radiographs, as assessed independently by two fellowship-trained orthopedic surgeons blinded to the clinical outcome. Union time was measured from the day of surgery to the date when radiographic and clinical criteria were fulfilled.

We recorded the age, gender, weight, duration of fracture and time to union on a pre-designed data entry form. Results were analyzed using SPSS version 22 (IBM Corp, Armonk, NY). Continuous variables were described as mean±standard deviation. Categorical variables were presented as frequencies and percentages. We performed stratification for age, gender, weight, and duration of fracture. An independent t-test was applied to compare mean union times. A p value of 0.05 or less was considered statistically significant.

## Results

A total of 68 patients were included in the final analysis. The demographic and clinical characteristics of study participants are presented in Table 1. The mean age was 8.03 years with a standard deviation of 2.16 years. The majority of the patients (n=37, 54.41%) belonged to the age group of one to seven years. There were 40 (58.82%) men and 28 (41.18%) women. The mean weight of the patients was 29.03 kg with a standard deviation of 3.12 kg.

**Table 1:** Patient Demographics and Clinical Characteristics (n=68) Data are presented as mean±standard deviation (SD) for continuous variables and as number (n) and percentage (%) for categorical variables.

Characteristic	Value
Age (years), Mean±SD	8.03±2.16
Age Group, n (%)	
1-7 years	37 (54.41%)
8-15 years	31 (45.59%)
Gender, n (%)	
Male	40 (58.82%)
Female	28 (41.18%)
Duration of Fracture (days), Mean±SD	4.22±1.43
Weight (kg), Mean±SD	29.03±3.12

The primary outcome was the mean duration of union for tibial shaft fractures treated with TEN. This was found to be 12.41 weeks with a standard deviation of 2.30 weeks.

Stratification analysis was conducted based on different influencing factors of the union time. Table 2 presents these results. We found statistical difference in terms of age distribution. Older patients, aged eight to 15 years, achieved union significantly sooner. The mean union time was 11.61 weeks (SD=1.15 weeks). The younger age group, one to seven years, had a mean union time of 13.08 weeks with a standard deviation of 2.78 weeks. The p value was 0.008. On the other hand, we did not observe a statistically significant difference in the union time according to sex, fracture to surgery duration or weight of the patient.

**Table 2:** Stratification of Mean Duration of Union (Weeks) Data are presented as mean ± standard deviation (weeks). Statistical comparison was performed using an independent samples t-test, with the t-value and p-value reported for each variable.

Stratification Variable	Category	Mean Union (weeks)	SD	p-value	t-value
Age Group	1-7 years	13.08	2.78	0.008	2.73
	8-15 years	11.61	1.15		
Gender	Male	12.6	2.38	0.424	0.8
	Female	12.14	2.19		
Weight	≤30 kg	12.19	2.44	0.413	0.82
	>30 kg	12.66	2.15		
Fracture to Surgery	0-3 days	12.5	2.32	0.829	0.22
	4-6 days	12.37	2.31		

## Discussion

The results of our study illustrated TEN to be a very successful intervention in children with tibial shaft fractures. The mean time to union was 12.41 weeks, consistent with the range of outcomes reported in contemporary studies, which generally span 10 to 13 weeks [7,8,10]. This method works based on a biomechanical principle to ensure stable and elastic fixation. The symmetrical pre-bent nails create a three-point pressure construct. It preserves alignment while allowing advantageous micromotion. This micromotion is an important biological factor in that it leads to solid periosteal callus. It allows a more physiologic and faster secondary bone healing as opposed to primary healing by rigid compression plating [4,11].

Our study provided important data regarding the impact of patient age on the time frame of fracture union. Children in the younger cohort, aged one to seven years, exhibited a markedly longer mean union time of 13.08 weeks; however, older children who were eight to 15 years old had lesser mean union time of 11.61 weeks. This inconsistency may be due to different biological and behavioral characteristics in younger children. Their periosteum is thicker and more osteogenic with quicker callus formation, but the immature initial callus may have less organization. It may be due to a longer consolidation period needed for mechanical strength [12]. In addition, partial weight bearing may be poorly tolerated by younger patients. This may subtly affect the preferred mechanical regimen for healing. Anatomic location may also contribute to this discrepancy; fractures closer to the physis tend to heal more rapidly than those farther away, and a higher proportion of such fractures in the older cohort could partly explain their shorter union time. Variations in intraoperative complexity or postoperative complications between groups may similarly influence outcomes, although these factors were not specifically assessed in the present analysis [4]. This age related variation in healing trajectory should be taken into account when counseling families regarding expected recovery time. 

A systematic review and meta-analysis by Fanelli et al. on flexible intramedullary nailing for pediatric tibial fractures provided evidence of its efficacy and low major complications [13]. Moreover, when directly compared to alternative surgical methods, TEN is consistently associated with better results. A comparative study by Pennock et al. reported that radiographic end results for patients treated by TEN were no different compared to plating. They also trended toward a shorter operative time and much earlier return of function [14]. The absence of major complications in our series, including nonunion and deep infection, further supports the safety and efficacy of this technique when performed with meticulous surgical technique.

It is necessary to recognise the controversy from the literature. Some studies in the literature suggest disadvantages of TEN compared to other procedures. For example, some studies suggest excess risk of malalignment, especially valgus and procurvatum deformities, in length-unstable fractures with plate osteosynthesis when compared to intramedullary nail fixation or external fixation [15]. Other studies describe a higher rate of implant-associated irritation leading to hardware removal in the TEN group which may be an important factor for families [16]. In the search of strategies to reduce these adverse events, though conflicting literature those two reports clearly, emphasize the importance of an accurate patient selection (including fracture pattern and location) and technical handling.

The present study has some limitations. Its descriptive, single-centre design with no comparative control group preclude definitive causal inferences being made. The second post-operative review time may be too short to detect late complications such as implant-related irritation needing removal. Rigorous, prospective, randomized controlled trials of TEN versus other techniques (plate fixation or modern external fixation modalities) in such age group would be a higher-level proof. Nevertheless, our findings remain clinically relevant, as they provide real world evidence supporting the effectiveness of this practice.

## Conclusions

This study conclusively finds that TEN is a simple, effective, and minimally invasive procedure for managing unstable tibial shaft fractures in children. It yields a predictable and favourable union time, with a low risk of significant complications. The significantly longer healing time observed in younger children is a critical factor for preoperative planning and patient counselling. On the basis of our findings, we strongly advocate the use of TEN as an established and valid surgical option for these types of injuries in children. TEN contributes to reduced morbidity and facilitates a swift return to normal childhood activities.
